# AI-Assisted Cardiovascular Risk Assessment by General Practitioners in Resource-Constrained Indonesian Settings Using a Conceptual Prototype: Randomized Controlled Study

**DOI:** 10.2196/73131

**Published:** 2025-11-25

**Authors:** Anindya Pradipta Susanto, David Lyell, Bambang Widyantoro, Dafsah Arifa Juzar, Anwar Santoso, Shlomo Berkovsky, Farah Magrabi

**Affiliations:** 1 Centre for Health Informatics, Australian Institute of Health Innovation, Macquarie University, 75 Talavera Road, Sydney, 2113, Australia, 61 423309268; 2 Medical Technology Cluster, Indonesian Medical Education and Research Institute, Faculty of Medicine, Universitas Indonesia, Jakarta, Indonesia; 3 Department of Medical Physiology and Biophysics, Faculty of Medicine, Universitas Indonesia, Jakarta, Indonesia; 4 Department of Cardiology and Vascular Medicine, Faculty of Medicine, National Cardiovascular Centre Harapan Kita Hospital Jakarta, Universitas Indonesia, Depok, Indonesia

**Keywords:** artificial intelligence, automation, clinical decision-making, clinical decision support, heart disease risk factors, risk assessment

## Abstract

**Background:**

Preventive strategies integrated with digital health and artificial intelligence (AI) have significant potential to mitigate the global burden of atherosclerotic cardiovascular disease (ASCVD). AI-enabled clinical decision support (CDS) systems increasingly provide patient-specific insights beyond traditional risk factors. Despite these advances, their capacity to enhance clinical decision-making in resource-constrained settings remains largely unexplored.

**Objective:**

We conducted a randomized controlled study to assess the effect of AI-based CDS on 10-year ASCVD risk assessment and management in primary prevention.

**Methods:**

In a 3-way, within-subject randomized design, doctors completed 9 clinical vignettes representative of primary care presentations in a resource-constrained outpatient setting. For each vignette, participants assessed 10-year ASCVD risk and made management decisions using a conceptual prototype of AI-based CDS, automated CDS, or no decision support. The conceptual prototype represented contemporary risk calculators based on traditional machine learning models (eg, random forest, neural networks, logistic regression) that incorporate additional predictors alongside traditional risk factors. Primary outcomes were correct risk assessment and patient management (prescription of aspirin, statins, and antihypertensives; referral for advanced examinations). Decision-making time and perceptions about AI utility were also measured.

**Results:**

In total, 102 doctors from all 7 geographical regions of Indonesia participated. Most (n=85, 83%) participants were 26‐35 years of age, and 57 (56%) were male, with a median of 6 (IQR 4.75) years of clinical experience. AI-based CDS improved risk assessment by 27% (*χ*^2^_2_ (n=102)=48.875, *P*<.001) when compared to unassisted risk assessment, equating to 1 additional correct risk classification for every 3.7 patients where doctors used AI (number needed to treat=3.7, 95% CI 2.9-5.2). The prescription of statins also improved by 29% (*χ*^2^_2_ (n=102)=36.608, *P*<.001). In pairwise comparisons, doctors who were assisted by the AI-based CDS correctly assessed significantly more cases (z=−5.602, n=102, adjusted *P*<.001) and prescribed the appropriate statin more often (*z*=−4.936, adjusted *P*<.001, medium effect size *r*=0.35) when compared with the control. AI-assisted cases required less time (estimated marginal means 63.6 s vs 72.8 s, *F*_2, 772.8_=5.710, *P*=.003). However, improvements in the prescription of aspirin and antihypertensives did not reach statistical significance (*P*=.08 and *P*=.30, respectively). No improvement was observed in referral decisions. Participants generally viewed AI-based CDS positively, with 81 (79%) agreeing or strongly agreeing that they would follow its recommendations and 82 (82%) indicating they would use it if given access. They believed CDS could enhance the efficiency of risk assessment, particularly in high-volume primary care settings, while noting the need to verify AI recommendations against clinical guidelines for each patient.

**Conclusions:**

Improvements in risk assessment and statin prescription, coupled with reduced decision-making time, highlight the potential utility of AI in ASCVD risk assessment, particularly in resource-constrained settings where efficient use of health care resources and doctors’ time is crucial. Further research is needed to ascertain whether improvements observed in this online study translate to real-world low-resource settings.

## Introduction

Atherosclerotic cardiovascular disease (ASCVD) remains one of the top global health concerns [1]. It is estimated that 80% of ASCVD cases are preventable [2]. Strategies to identify and target people at higher risk are therefore crucial to reducing the global disease burden, especially in resource-constrained settings with limited access to medical specialists and advanced equipment [3,4].

Primary care doctors play an important role in preventing ASCVD by assessing and managing cardiovascular risk factors. Risk assessment is centered on routine screening of asymptomatic adults 40‐75 years of age to determine the risk of heart disease or stroke in the next 10 years, allowing for more focused primary preventive interventions. The intensity of interventions must align with the patient’s individual risk, optimizing anticipated benefits while minimizing potential harm from overtreatment and allocating limited resources appropriately [5]. Doctors often use clinical decision support (CDS) tools to calculate ASCVD risk and guide shared decision-making for preventive management [6,7]. In resource-constrained settings however, medical records are predominantly maintained on paper, and doctors use CDS on mobile devices [8] (AP Susanto, PhD, et al; unpublished data, August 2024). This situation warrants an automated process to enter patient data into the CDS, as manual data entry is prone to errors, especially under high patient loads (AP Susanto, PhD, et al; unpublished data, August 2024).

Recently, CDSs are being embedded with artificial intelligence (AI) [9], specifically machine learning (ML) models that can accommodate patient-level precision beyond traditional risk factors. These AI-based CDSs provide new opportunities to improve risk assessment in poorly served patient subgroups and consider nontraditional risk factors, such as psychosocial factors, or inflammation [10,11]. While ML models have demonstrated superior performance compared to statistics-based ASCVD risk calculators [12], their impact on enhancing clinical decisions, which is an essential precursor for realizing benefits in care delivery and patient outcomes, remains unknown [13]. In a recent review of AI tools in routine care, only 7 studies examined clinical decision-making, none of the 86 randomized controlled trials published in the last 6 years focused on risk assessment for cardiovascular disease (CVD), and only 4 were conducted in resource-constrained settings [14].

We sought to evaluate whether AI-based CDS can improve clinical decisions for the primary prevention of ASCVD in Indonesia, where CVD accounts for the highest health care expenditure [15]. The high prevalence of CVD risk in Indonesia (29.2%) underscores the need to prioritize preventive services [16]. Accurate risk assessment is fundamental to preventive care, yet it remains a persistent challenge, especially among high-risk rural residents who are less likely to receive such care. Consequently, many individuals at elevated risk are not identified, whereas others without risk are unnecessarily subjected to preventive interventions. A randomized controlled study was conducted to assess the effect of AI-based CDS on 10-year ASCVD risk assessment and management, using clinical vignettes with a conceptual prototype of a CDS. Clinical vignettes or patient cases offer greater study control to quantify the effects of AI-based CDS on decision-making before costly and potentially disruptive clinical deployment, allowing for evaluation without putting real patients or doctors at risk [17,18]. The patient cases were based on our previous work, which examined the sociotechnical context of CVD risk assessment in resource-constrained settings with limited access to medical specialists and advanced equipment (AP Susanto, PhD, et al; unpublished data, August 2024). Acute coronary syndrome caused by atherosclerosis was confirmed as the most common CVD challenge, requiring primary prevention to alleviate the disease burden. In this study, we compared AI-based CDS against the current approach to risk assessment with either no CDS or automated CDS, whereby patient data were automatically populated into the CDS. The technology acceptance of CDS was also examined.

## Methods

### Setting and Participants

In total, 102 doctors participated in the online study between November and December 2023. Participants were recruited by publishing a call for volunteers via the Indonesian Heart Association at 5 training centers located in different geographical regions. Doctors who were working in general practice or enrolled in a cardiology training program were eligible to participate. The study invitation was also distributed to potential participants via email and WhatsApp (Meta Platforms).

### Design

**Figure 1.**
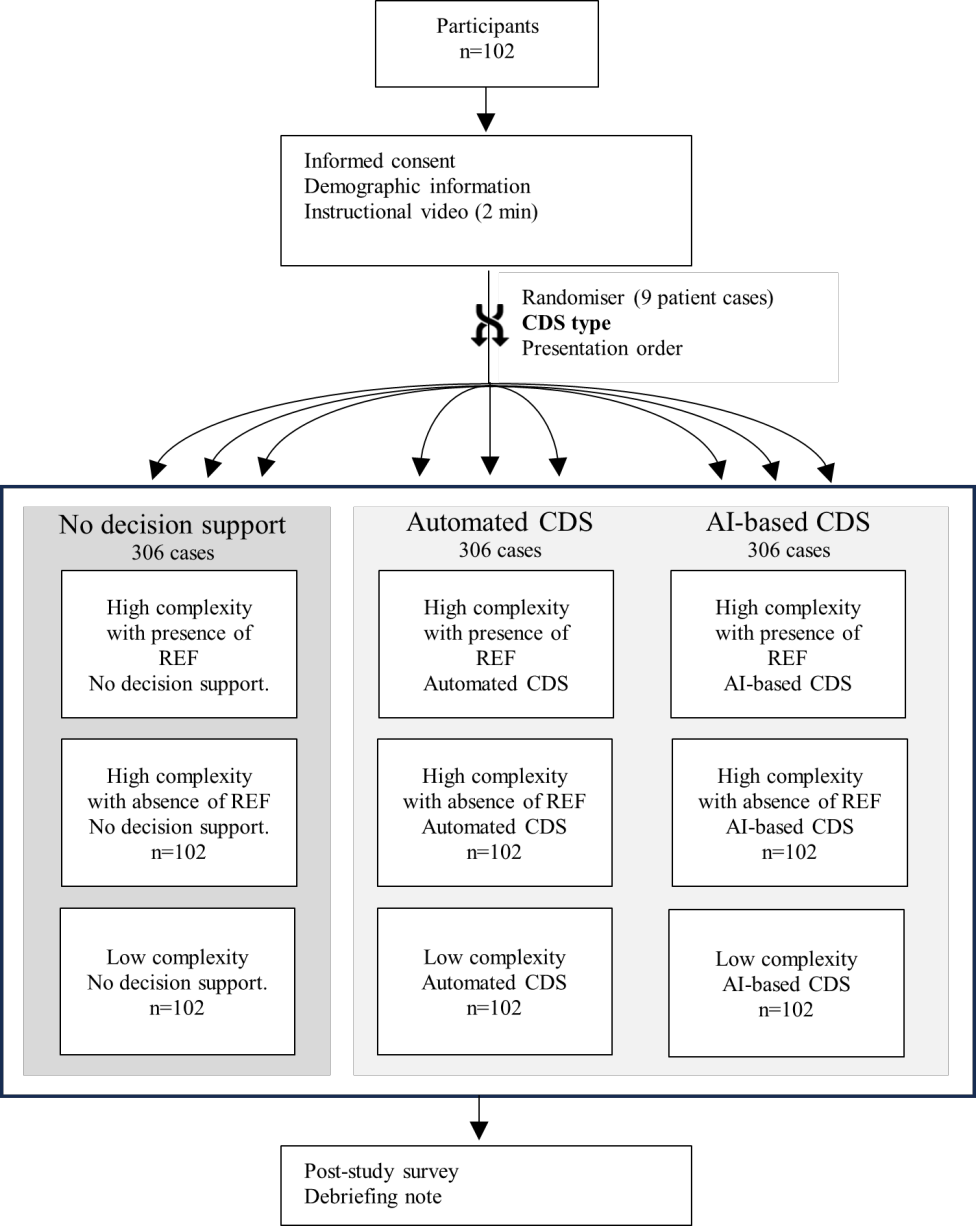
Design and procedure. Participants completed 9 patient cases. AI: artificial intelligence; CDS: clinical decision support; REF: risk-enhancing factor.

We performed a 3-way, within-subject randomized controlled study to evaluate the effect of CDS type: standard care with no CDS, automated CDS, and AI-based CDS (see Figure 1 and the *Intervention* section for details). To simulate variation in the clinical context, each CDS type was provided with 3 patient cases with different case complexity levels: low, high, and high with risk-enhancing factors (REFs) based on the literature [19,20]. This design provided 9 patient cases related to primary prevention in an outpatient setting for adults aged 40-75 years without a history of ASCVD (AP Susanto, PhD, et al; unpublished data, August 2024). Low-complexity cases only contained information about the traditional risk factors, including age, sex, race, blood pressure, cholesterol level, history of diabetes, smoking status, and previous medications including antihypertensives, statin, and aspirin. High-complexity cases included the presence (3 cases of high complexity with REFs) or absence (3 high-complexity cases) of REFs such as family history of premature heart attack and obesity, on top of the traditional risk factors.

The study was deployed using Gorilla.sc, a platform for conducting online studies, which was customized to display patient cases and CDS interventions [21]. The allocation of cases to the different CDS types and the presentation order were randomized so that each case would appear evenly across the 3 CDS types. All randomizations were set equal (1:1) and performed by the Gorilla software (v2; Cauldron Science).

### Intervention

**Figure 2.**
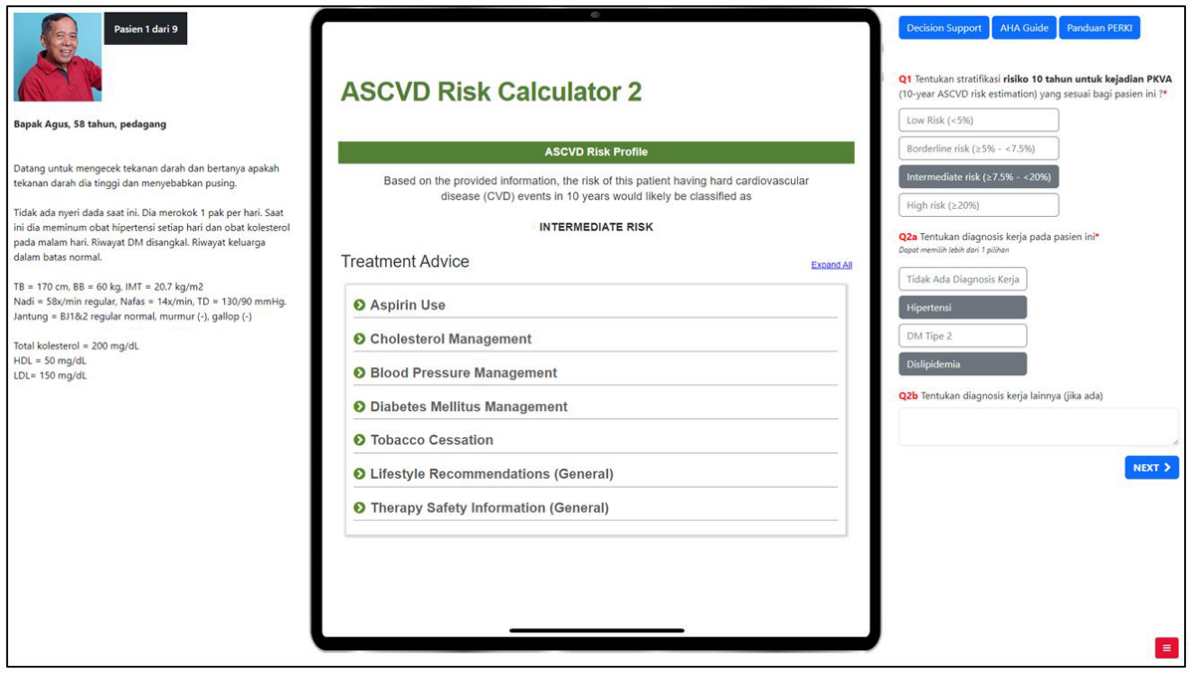
The online interface for the study was divided into 3 panes. The left side presented the patient case. A mock-up of a mobile device interface in the middle pane showed the clinical decision support (CDS) intervention and could be switched to display clinical guidelines by clicking the buttons on the top right. This case shows ASCVD Risk Calculator 2, which corresponds to the conceptual prototype of the artificial intelligence–based CDS. Participants were asked to review the case and respond to questions in the right pane. ASCVD: atherosclerotic cardiovascular disease.

The CDS type provided to participants was manipulated across 3 conditions as follows:

1. No decision support, that is, the current approach to risk assessment served as the control condition. Only the information present in the case was shown (see Multimedia Appendix 1).
2. Automated CDS: This was designed to augment the current approach to risk assessment whereby patient data were automatically populated, and the CDS output was displayed to participants, streamlining screening for doctors with high patient load. The automated CDS presented the 10-year ASCVD risk level, risk score (in %), and treatment advice in English. It was based on the publicly available ASCVD Risk Estimator Plus calculator from the American College of Cardiology [20], which is widely accepted in Indonesia. Risk is calculated using Pooled Cohort Equations, a conventional statistical method based on traditional risk factors. Although the display of the original calculator was not altered, the logo was concealed (see Multimedia Appendix 1).
3. AI-based CDS: This was designed to represent contemporary risk calculators based on traditional ML models (eg, random forest, neural networks, logistic regression) that account for additional risk factors on top of the traditional risk factors and have been shown to perform better in risk discrimination compared to traditional risk estimators [12]. Here, a conceptual prototype of an AI-based CDS was constructed similar to previous studies examining doctors’ perceptions about ML-based risk calculators [22]. The AI-based CDS presented the 10-year ASCVD risk level and treatment in English but did not provide a risk score (in %). The CDS output, that is, the risk assessment and treatment advice for each case, was constructed by a general practitioner (GP; APS), then scored independently by 2 consultant cardiologists (DAJ and AS), and reviewed by a third consultant cardiologist (BW) based on their clinical experience in managing patients with CVD in Indonesia. All disagreements were resolved by consensus in a workshop (see Figure 2 and Multimedia Appendix 1).

Blinding was implemented to minimize placebo effects. The allocation of patient cases to CDS type was according to 9 pregenerated sequences, created so that cases were allocated evenly across each of the 3 conditions. Participants were allocated to sequences at the time of enrollment using balanced randomization, with sequences presented in random order. All conditions were displayed using an identical user interface. While participants were able to distinguish between the control (no decision support) and intervention conditions, they were blinded to the intervention conditions. The automated CDS and AI-based CDS were generically coded as “Risk Calculator 1” and “Risk Calculator 2,” respectively, with an identical presentation (Figure 2). We did not specify which tool harnessed AI to minimize placebo effects that could influence doctors’ performance [23]. In all conditions, participants could access 2 clinical guidelines on ASCVD prevention [20,24], commonly referred to by Indonesian doctors [25].

### Patient Cases

The patient cases were based on the clinical guidelines for the primary prevention of CVD [20,24], covering typical presentations of asymptomatic adults 40‐75 years of age in an outpatient setting. Participants were presented with 9 patient cases and asked to assess the 10-year risk of ASCVD and provide the most appropriate patient management. As shown in Figure 2, each case included a brief patient history, physical examination, and simple laboratory results commonly available in resource-constrained clinical settings (Multimedia Appendix 1). The study setup and the CDS interface were designed to emulate access on a mobile device, which is a likely real-world implementation of such tools in resource-constrained settings [8].

Cases and questions were presented in Bahasa Indonesia to ensure clarity. The cases were developed by a GP (APS) with advice from a senior cardiologist consultant (BW). Risk factors, risk assessment, and patient management in each case were coded by APS and reviewed by BW; disagreements were resolved by consensus. The gold standard for risk assessment was determined based on the clinical guidelines, and cases with REFs were reclassified based on clinical relevance. Cases and gold standard responses for correct risk assessment and patient management were independently reviewed by an expert panel of 2 senior cardiology consultants (DAJ and AS) to ensure clinical relevance. Disagreements were resolved by consensus via a finalization workshop.

### Procedure

Participants self-enrolled in the online study. The study URL was provided in the invitation, and participants were asked to access the study from a laptop or desktop computer with an internet connection at their convenience. After informed consent, participants provided demographic information and watched an instructional video (2 min) explaining the study task and CDS interventions, orienting participants with the study user interface.

Participants were also shown how to view clinical guidelines that were accessible online. At the end of the video, participants had the opportunity to explore the user interface and CDS interventions with a demonstration case and could repeat the demonstration case once.

Participants were randomly allocated a pregenerated sequence of 9 cases. Cases that were allocated to the automated CDS (blinded as “Risk Calculator 1”) and AI-based CDS (“Risk Calculator 2”) conditions presented the risk level and patient management. Control cases displayed “No Decision Support” where CDS would not be displayed in the mobile device interface.

Participants were instructed to complete all 9 cases to the best of their clinical judgment in a single, uninterrupted session, and no time limits were imposed. After completing the cases, participants responded to a poststudy survey based on the technology acceptance model (TAM) [26]. They were then provided with a debriefing note and thanked for their participation. The procedure was pilot-tested by 2 doctors and refined based on their feedback.

### Outcome Measures

To assess the effects of AI-based CDS on risk assessment and patient management, the following outcome measures were examined.

#### Risk Assessment

The response was multiple choice and automatically scored as correct or incorrect against the validated gold standard response for the cases. Given the case, participants determined the 10-year ASCVD risk assessment, defined as a chance of having major CVD events (eg, heart disease or stroke) in the next 10 years. There were 4 levels of risk: low risk (<5%), borderline risk (≥5% to <7.5%), intermediate risk (≥7.5% to <20%), and high risk (≥20%). We counted the number of cases correctly assessed.

#### Patient Management

As with the risk assessment, the response for patient management was multiple choice and automatically scored as correct or incorrect against the validated gold standard response for the case. Given the case and risk classification, there were 4 measures of patient management for primary prevention: whether the doctor discussed the risk level or score and prescribed aspirin, statins, antihypertensives, and referred patients for advanced examinations. We counted the number of cases correctly managed as follows.

1. Aspirin prescription: Participants chose between not prescribing or prescribing 80‐100 mg of aspirin.
2. Statin prescription: Participants choose 1 of 4 options: (1) no statin therapy, (2) low-intensity statin (eg, simvastatin 10‐20 mg), (3) moderate statin intensity (eg, simvastatin 20‐40 mg or atorvastatin 10‐20 mg), or (4) high-intensity statin (eg, atorvastatin 40‐80 mg or rosuvastatin 20‐40 mg).
3. Antihypertensive prescription: Participants chose between discussing and prescribing antihypertensive medications or not.
4. Advanced examination referral: Participants chose between referring and not referring patients to a facility located 1‐3 hours away for advanced examinations such as treadmill stress test [27], computed tomography calcium score [28], or computed tomography coronary angiography [29].

#### Decision-Making Time

Decision-making time was automatically measured from when the case was first presented until the responses for risk assessment and diagnosis were submitted.

#### Technology Acceptance

Participant perceptions about the potential utility of AI for ASCVD risk assessment in resource-constrained settings were examined using mixed methods [26]. In the quantitative component, we examined perceptions about the automated CDS and AI-based CDS using the TAM, including the usefulness (2 items), ease of use (1), and behavioral intention of CDS usage (2) using a 7-point Likert scale ranging from (“strongly disagree”) to 7 (“strongly agree”). Participants’ free-text responses to “comments about CDS” were coded into different factors of the TAM by APS and reviewed by FM.

We also measured effects of case complexity, diagnosis, choice of specific antihypertensive regimen, and justification for referrals; these analyses will be reported separately.

### Statistical Analysis

We estimated that 61 participants would be required to detect a 39% or greater difference (2-tailed) in decisions with and without CDS with 90% power and at *P*<.05 [30]. All measures for risk assessment and patient management were non-normally distributed, and therefore, the effects of CDS were tested by using the Friedman test to compare multiple repeated measures of cases correctly assessed and managed across CDS types. Subsequent CDS pairwise comparisons with Bonferroni-adjusted *P* values and effect sizes were performed between the CDS type pair.

Decision-making time was analyzed by a multilevel model with outliers removed (see Multimedia Appendix 2) [31,32]. The model included a random intercept for each participant, controlling the nested nature of data. Predictors assessed for inclusion in the model were CDS type and case complexity. A stepwise backward elimination method was used for predictor selection, where all predictors and interactions were entered into the model. Models were estimated using maximum likelihood, and fit was evaluated. Predictors that significantly improved model fit were retained.

For technology acceptance, the median of all participant scores was calculated for each item. To assess the internal consistency of the TAM factors in the questionnaire, we calculated the mean of the items for each factor and Cronbach α [26].

All statistical analyses were undertaken using the SPSS software (v27; IBM Corp). Reporting was guided by the health care simulation research extensions of the CONSORT (Consolidated Standards of Reporting Trials) and STROBE (Strengthening the Reporting of Observational Studies in Epidemiology) statements (see Checklist 1) [33].

### Ethical Considerations

This study was approved by the Macquarie University Human Research Ethics Committee (reference: 520221032837485; project: 10328) and Faculty of Medicine Universitas Indonesia Ethics Committee (KET-586/UN2.F1/ETIK/PPM.00.02/2022). Participant consent was obtained via an online participant information sheet and consent form in accordance with approved protocols. Participants were offered an IDR 500,000 (US $32) e-commerce voucher for their contribution. No personally identifying information was used for analysis and reporting. Patients presented in the cases were hypothetical, biographical information was generated solely for the purposes of this study, and patient photos were produced by generative AI (Dall-E by OpenAI in October 2023). This study was not prospectively registered, as it was conducted using a conceptual prototype with clinical vignettes rather than real patients. It was retrospectively registered with the Australian New Zealand Clinical Trials Registry (ACTRN12625001168448 [34]).

## Results

### Demographic Characteristics

**Figure 3.**
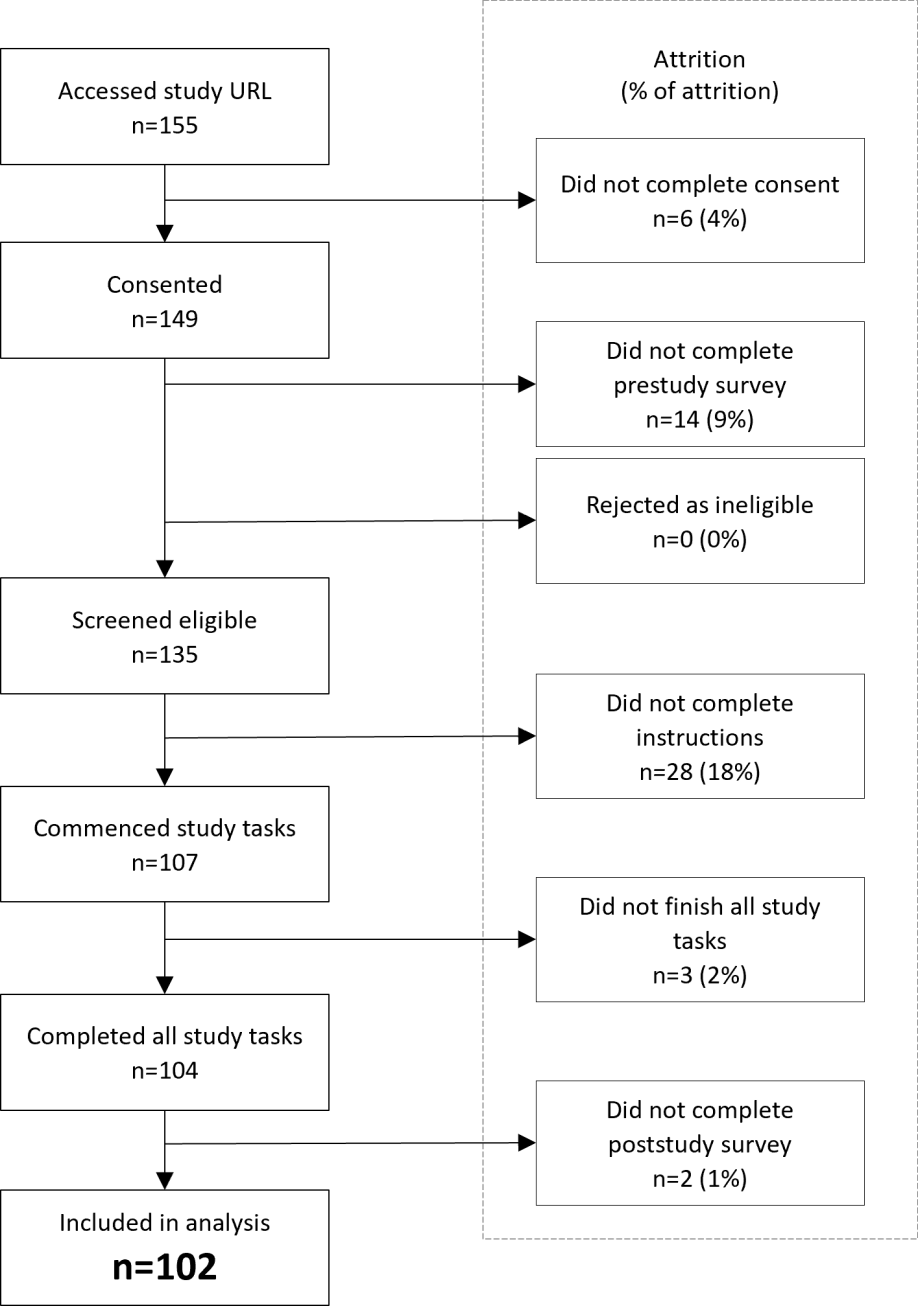
CONSORT (Consolidated Standards of Reporting Trials) diagram showing the flow of participants through the study.

**Figure 4.**
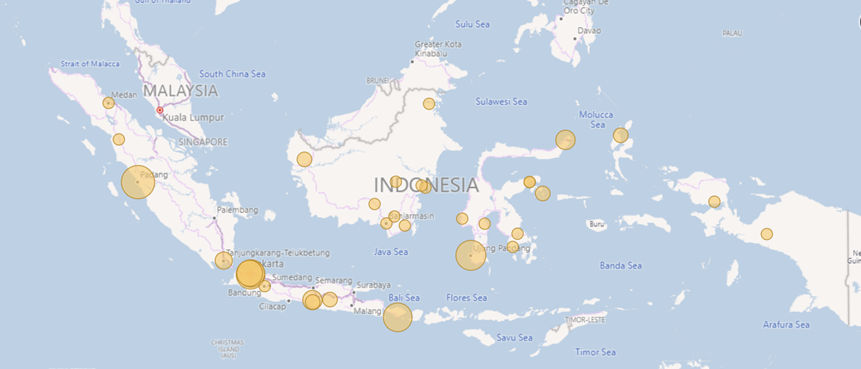
Study participants were distributed across 33 cities or regions in 21 provinces, representing all 7 geographical units of Indonesia. A map of Indonesia was visualized by Power BI (Microsoft Corporation).

In total, 102 Indonesian doctors participated in the online study (Table 1; Figure 3). The participants were distributed across all 7 geographical units of the Indonesian archipelago (Figure 4). Most (n=57, 56%) identified as male and were aged 26‐35 years (n=85, 83%), with a median of 6 (IQR 4.75) years of clinical experience. Of these, 63 (62%) were enrolled in a 4-year graduate cardiology residency training program, which requires 1‐5 years of experience as a GP following graduation from undergraduate medical school and is usually undertaken in a remote area.

**Table 1.** Demographic characteristics of participants (n=102 doctors).

Participant characteristics	Participants, n (%)	
Gender		
Male	57 (56)	
Female	45 (44)	
Age (years)		
<26	2 (2)	
26‐30	48 (47)	
31‐35	37 (36)	
36‐40	5 (6)	
41‐45	3 (4)	
46‐50	1 (1)	
51‐55	0 (0)	
>55	3 (5)	
Clinical role seniority		
GP^a^	38 (37)	
GP cardiology resident stage 1	44 (43)	
GP cardiology resident stage 2 and 3 or final	19 (19)	
Workplace^b^		
Hospital type A	58 (57)	
Hospital type B	2 (2)	
Hospital type C	3 (3)	
Hospital type D	2 (2)	
*Puskesmas* (community health center)	11 (11)	
Clinic (primary or main)	23 (23)	
Solo practice	3 (3)	
Geographical unit		
Jawa	36 (35)	
Sulawesi	23 (23)	
Sumatera	19 (19)	
Kalimantan	10 (10)	
Bali and Nusa Tenggara	10 (10)	
Maluku	2 (2)	
Papua	2 (2)	
Self-reported English reading level		
Proficient	28 (27)	
Intermediate	52 (51)	
Basic	21 (21)	

a GP: general practitioner.

b Medical services are provided by provincial or national general hospitals (class A and B) as well as city or district hospitals (class C and D) [35].

### AI-Based CDS Improved Risk Assessment

We examined the effect of CDS type on risk assessment by comparing cases correctly assessed across the 3 study conditions: standard care with no CDS (control), automated CDS, and AI-based CDS (Table 2). The analysis revealed a statistically significant difference in risk assessment by CDS type (Table 3: *P*<.001). In pairwise comparisons, a significantly higher number of cases were correctly assessed when doctors were assisted by the AI-based CDS compared to the control (*z*=−5.602, n=102, adjusted *P*<.001, medium effect size *r*=0.39) and the automated CDS (z=−4.901, n=102, adjusted *P*<.001, medium effect size *r*=0.34). However, the improvement in risk assessment with the automated CDS compared to the control was nonsignificant (*z*=−0.700, n=102, adjusted *P*>.99).

**Table 2.** Number of patient cases correctly assessed and managed.^a^

Outcome measures	Control	Intervention/CDS^b^ type	
	No CDS	Automated CDS	AI^c^-based CDS
Risk assessment, n (%)^d^	135 (44.1)	140 (45.8)	217 (70.9)
Patient management, n (%)^d^			
Aspirin prescription	206 (67.3)	220 (71.9)	192 (62.7)
Statin prescription	108 (35.3)	185 (60.5)	197 (64.4)
Antihypertensive prescription	245 (80.1)	249 (81.4)	258 (84.3)
Referral for advanced examinations	199 (65.0)	208 (68.0)	175 (57.2)

a Each CDS type was provided with 3 patient cases with different case complexity levels to cover variation in clinical contexts.

b CDS: clinical decision support.

c AI: artificial intelligence.

d “n (%)” refers to the number and percentage of cases correctly assessed and managed out of 306 cases.

**Table 3.** Impact of artificial intelligence (AI)–based clinical decision support (CDS) on cardiovascular disease (CVD) risk assessment, patient management, and decision-making time.

Outcome measures	Control	Intervention/CDS type		Friedman test and pairwise comparison effect size
	No CDS	Automated CDS	AI-based CDS	
Risk assessment, median (IQR)	1 (1-2)	1 (1-2)	3 (1-3)	*χ*^2^_2_^a^ (n=102)=48.875, *P*<.001^b^; control versus AI *r*^c^=0.39; AU^d^ versus AI *r*=0.34
Patient management, median (IQR)				
Aspirin prescription	2 (2-3)	2 (2-3)	2 (1-3)	*χ*^2^_2_ (n=102)=6.428, *P*=.08
Statin prescription	1 (0‐2)	1 (1-2)	2 (1-3)	*χ*^2^_2_ (n=102)=36.608, *P*<.001^b^; control versus AI *r*=0.35; control versus AU *r*=0.28
Antihypertensive prescription	3 (2-3)	3 (2-3)	3 (2-3)	*χ*^2^_2_ (n=102)=2.378, *P*=.30
Referral for advanced examinations	2 (1-3)	2 (1-3)	2 (1-2)	*χ*^2^_2_ (n=102)=7.066, *P*=.03^b^; no significant differences in pairwise comparison
Decision-making time^e^, EMM^f^ (95% CI) in seconds	72.8 (63.9‐82.9)	62.6 (55.0‐71.3)	63.6 (55.9‐72.3)	*F*^g^_2, 772.8_=5.710, *P*=.003

a Chi-square value by Friedman test.

b Indicates significant differences (*P*<.05) determined by Friedman test or multilevel model.

c Effect size for statistically significant difference in pairwise comparison.

d AU: automated CDS.

e Decision-making time was analyzed using the multilevel model for decision-making.

f EMM: estimated marginal mean.

g Ratio by multilevel model.

### Effects of AI-Based CDS on Patient Management Were Mixed

We observed a mixed effect of CDS type on the 4 patient management measures, including the prescription of aspirin, statin, antihypertensive medications, and referral for advanced examinations (Tables 2 and 3). While CDS type had a statistically significant effect on the prescription of statins (*P*<.001) and referrals (*P*=.03), its effect on the prescription of aspirin and antihypertensives was nonsignificant.

In pairwise comparisons, a significantly higher number of cases were prescribed with the appropriate statin intensity when doctors were assisted by the AI-based CDS (*z*=−4.936, adjusted *P*<.001, medium effect size *r*=0.35) and the automated CDS (*z*=−3.991, adjusted *P*<.001, small effect size *r*=0.28). For referrals, no differences were revealed in the pairwise comparison. While fewer cases were correctly referred with AI-based CDS compared to the control (*z*=0.230, adjusted *P*=.30) and automated CDS (*z*=2.135, adjusted *P*=.30), these differences were nonsignificant.

### AI-Based CDS Decreased Decision-Making Time

Cases assisted by the AI-based CDS (*P*=.02) and automated CDS *(P=.*006) took less time than the control (see Table 3 for estimated marginal means). There was no significant difference in the decision-making time between cases assisted by the automated CDS and AI-based CDS.

### Perceptions About CDS

We measured doctors’ perceptions about automated CDS and AI-based CDS using the TAM. Overall, doctors agreed with statements measuring the perceived ease of use, usefulness, and behavioral intention to use CDS, with a median score of 6 for all items (ie, “agree”). The value for Cronbach α for the survey was ≥.7, showing internal consistency within the factors. Of the 102 respondents, 81 (79%) agreed or strongly agreed to using recommendations from AI-based CDS and 82 (82%) agreed or strongly agreed to use it if given access. Perceptions about the accuracy of the AI-based CDS were largely positive, where 76 (76%) participants perceived their decisions on risk more accurate, while 6 (6%) somewhat disagreed with its accuracy.

**Figure 5.**
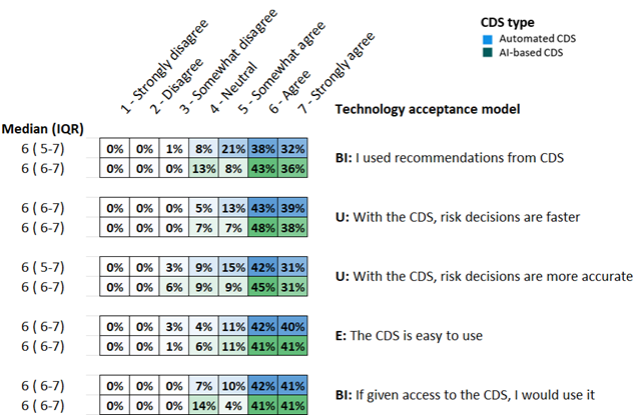
Heat map of 5 items assessing the technology acceptance in 102 doctors. Values are presented as relative frequencies (n=102). Technology acceptance model factors: U: perceived usefulness (α_AU_=.749 and α_AI_=.736); E: perceived ease of use; BI: behavioral intention to use (α_AU_=.711 and α_AI_=.873). AI: artificial intelligence; AU: automated CDS; CDS: clinical decision support.

For the automated CDS, 72 (70%) agreed or strongly agreed to using its recommendations, and 85 (83%) agreed or strongly agreed to using it if given access. Perceptions about the accuracy of the automated CDS were largely positive, where 75 (73%) participants perceived their decisions on risk were more accurate, while 3 (3%) somewhat disagreed with its accuracy (Figure 5).

Qualitative comments highlighted the overall usefulness of both CDS in providing comprehensive patient management (Textbox 1). Doctors believed that CDS had the potential to improve the efficiency of risk assessment and decision-making, particularly in primary care settings where patient volumes are high. While the AI-based CDS was perceived to be more accurate, the need to verify recommendations against clinical guidelines based on the patient’s actual condition was noted. The necessity for the AI-based CDS to offer explanations of its risk scores was also highlighted (Textbox 1, Doctor 014, Doctor 007). Automated input of patient data and the ability to work offline without an internet connection were identified as essential features for ease of use.

Textbox 1. **Participants’ comments about the automated and artificial intelligence (AI)–based clinical decision support (CDS). ASCVD: atherosclerotic cardiovascular disease; BI: behavioral intention to use; E: perceived ease of use; U: perceived usefulness.**

**Automated CDS (blinded as Risk Calculator 1)**

U, E: *The ASCVD Risk Calculator is quite good and comprehensive. It will also help streamline services, especially outpatient care such as clinics that require quick determination of patient risk and treatment. Hopefully, it will be easier to input data and can be used offline (without network/internet)* [Doctor 006]

U: *The ASCVD Risk Calculator is a tool that facilitates drawing conclusions regarding the risks faced by patients and accelerates our decision-making process regarding both treatment and further examinations.* [Doctor 083]

U: *Interesting and beneficial in making clinical decisions.* [Doctor 037]

U: *Very useful for quicker and concise clinical considerations, and can be adjusted between guidelines, patient conditions, and available medications.* [Doctor 098]

BI: *…overall, it is easily comprehensible and manageable. If given access, I will use it.* [Doctor 100]

**AI-based CDS (blinded as Risk Calculator 2)**

E: *Similar to Risk Calc 1, Risk Calc 2 also has several suggestions that should consider the actual patient condition in line with existing guidelines.* [Doctor 026]

U: *Compared to calculator 1, it feels more accurate. However, the explanations supporting the decisions feel lacking, so cross-checking with guidelines is still necessary.* [Doctor 014]

E: *This tool is less convincing because it doesn’t outline the specific risk factors present in the patient; it directly mentions their risk category instead.* [Doctor 007]

U: *It’s already good. It would be very helpful if it could be implemented in day-to-day clinical practice.* [Doctor 085]

U: *It is very helpful, especially in primary healthcare facilities such as Puskesmas, where there are many patients and quick and accurate decision-making is needed.* [Doctor 102]

## Discussion

### Principal Results and Implications

We found that use of AI-based CDS significantly improved 10-year ASCVD risk assessment and decreased decision-making time, with mixed effects on patient management. Overall, participants’ perceptions about AI assistance were favorable. The observed improvement indicates potential for AI-based CDS to improve routine screening for CVD risk factors in resource-constrained settings. The number needed to treat for benefit associated with AI-based CDS was around 4, indicating that for every 4 patients where doctors use AI-based CDS as the “treatment,” there will be 1 additional correct risk classification as the “outcome” (number needed to treat=3.7, 95% CI 2.9-5.2). Assuming daily primary care visits of 30-100 patients per doctor (AP Susanto, PhD, et al; unpublished data, August 2024), with 12% requiring ASCVD risk assessment [36], this amounts to an additional 1-3 accurate risk assessments per day, totaling 250-750 per year. Accordingly, these findings suggest the need for further research in a live clinical environment to determine whether improvements observed in the online study can be translated into real-world clinical settings.

For AI-based CDS to provide value, the decisions made by doctors must trigger some action or the CDS must provide actionable recommendations [37]. We measured patient management as an indicator of change in actionable care delivery, specifically the correct prescription of primary preventive medications (aspirin and statin), prescription of antihypertensive medications, and referral for advanced examinations. The findings indicated mixed effects on care delivery. Most importantly, AI-based CDS positively impacted the prescription of statins, showing that primary prevention of ASCVD could be improved by deploying AI tools to assist doctors. This is consistent with previous studies in Indonesia that identified gaps in awareness about the prescription of statins [25], which, when prescribed at an appropriate intensity based on individual risk assessments, can reduce the 10-year incidence of ASCVD, achieve lipid target levels, and minimize medication side effects for long-term safety [20].

Conversely, the effects of AI-based CDS in improving the prescription of aspirin and antihypertensive medications did not reach statistical significance. One possible explanation is that doctors may consider blood pressure independently of the ASCVD risk assessment and CDS recommendation when prescribing antihypertensives [38]. For aspirin, which had fewer cases of correct prescription, doctors appeared to ignore the CDS recommendations or did not see the guideline. Another possibility is a ceiling effect: the baseline correctness for these 3 measures in the control group (67.3% and 80.1%, respectively) was already substantially higher than for statins (35.3%), leaving less room for improvement. Consequently, doctors may have benefited less from the CDS when prescribing aspirin and antihypertensives, thereby limiting potential gains. Further research is needed to understand these effects.

We could not ascertain why AI-based CDS did not improve referral for advanced examinations. One possible explanation is that referrals were not included in the AI-based CDS recommendations, requiring doctors to infer the need for referral based on their knowledge of local access and clinical judgment. This is consistent with the ambiguity in the current national guidelines, which are silent about the referral of high-risk patients for advanced examination, possibly due to variations in local resources and policies. As such, this finding implies that the use of AI-based CDS, particularly for risk assessment, should be augmented with clear clinical consensus and local policy to enhance the actionability of recommendations to avoid mixed or negative effects on care delivery.

### Delivering Fast and Acceptable AI-Based CDS

We found that use of AI-based CDS reduced decision-making time by 12% compared to the control group, even though doctors with AI assistance received additional information and confirmed the risk assessment provided by the system. While the reduction in decision-making time is statistically significant, the 10-second difference may not be clinically meaningful. Nevertheless, this finding implied that decision-making assisted by AI remained faster than heuristic risk assessment without CDS. This suggests that using AI-based CDS decreases decision time, despite requiring doctors to review and confirm the AI output.

The observed effects on decision-making time are consistent with previous studies of AI in primary care. For instance, a randomized controlled study of cataract screening in a remote area suggested faster diagnosis when supported by AI-based CDS compared to doctors alone (2.79 min vs 8.52 min, *P*<.001) [39]. In another study simulating AI-based CDS for the treatment of depression, 40% of doctors indicated that using CDS would save time [40]. Thus, AI-based CDS may be especially valuable in high-volume, resource-constrained settings where clinician time is scarce (AP Susanto, PhD, et al; unpublished data, August 2024).

Doctors generally viewed CDS for ASCVD risk assessment positively, but they raised concerns about its accuracy, guideline adherence, and capacity to address patients comprehensively. Similar concerns were observed with the use of AI-based risk assessment for delirium [26]. In our study, most doctors found AI-based CDS useful for decision-making, and their intention to use it was reinforced by perceptions of its high accuracy. Conversely, an AI system for sepsis prediction was poorly rated due to late, less useful alerts and a lack of transparency in its “black-box” results [41]. Doctors who perceive AI as beneficial are more likely to adopt it in clinical practice.

### Safe Use of AI-Based CDS

Despite having access to highly accurate AI-based CDS, approximately 89 (29%) of patient cases were inaccurately assessed, as doctors failed to recognize accurate recommendations. This proportion appears to compare with 24 (24%) participants, who were neutral or somewhat disagreed with the accuracy of the AI in the TAM survey. Participant comments suggested that doctors sought explanations when their risk estimates diverged from CDS recommendations (Textbox 1). In such cases, doctors might have relied on their own risk estimates due to the AI’s lack of explanation.

**Figure 6.**
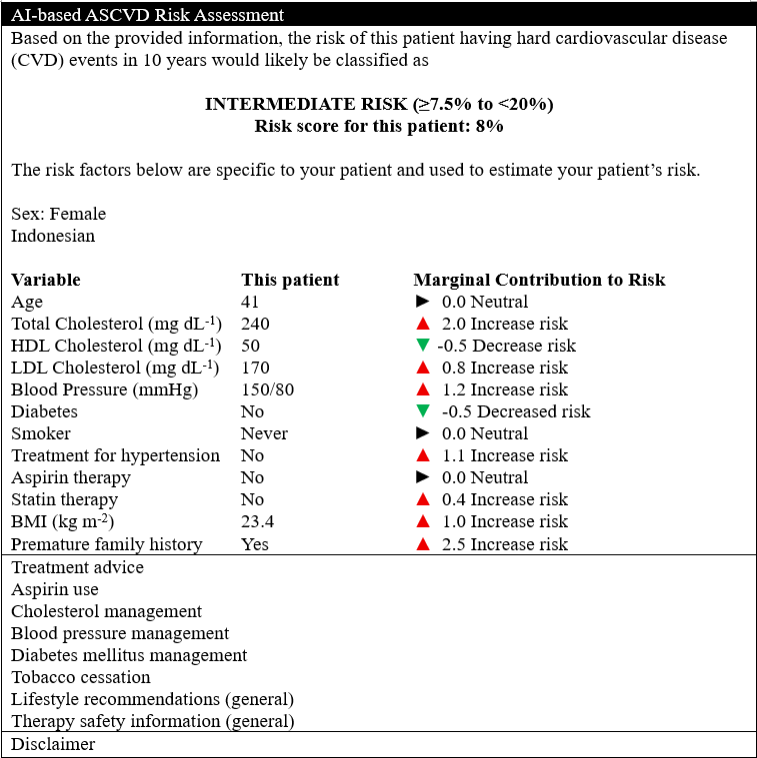
An example explanation to enhance future artificial intelligence (AI)-based clinical decision support for atherosclerotic cardiovascular disease (ASCVD) risk assessment in resource-constrained settings. Treatment advice and disclaimer notes are collapsed into pointers to maintain simplicity and emphasize the explanatory component.

For future development and validation, one way to increase doctors’ confidence and performance is for AI-based CDS to provide an explanation as demonstrated by the mock-up in Figure 6. Explanations could be model-agnostic and local, displaying risk factors, input variables, their marginal contributions, and risk scores, rather than only providing on risk level recommendations. Previous studies demonstrated that model-agnostic local explanations, which include individual risk factors, can help doctors comprehend, trust, and explain outputs to patients [22]. As doctors prefer simple visualizations and relevant clinical reasoning, AI should empower them to offer meaningful explanations to patients, facilitating shared decision-making.

Our results suggest that doctors need support through relevant clinical guidelines and training to make informed decisions about using and evaluating AI recommendations. Although participants had access to clinical guidelines, and comments emphasized their importance (Textbox 1), our findings are consistent with a previous study of prescribing decision support with 102 participants, where less accurate recommendations were accepted despite the availability of guidelines for verification [31]. Differently from high-resource settings, where verification can involve advanced examinations or medical specialists, in resource-constrained settings, verification mechanisms may be limited to simple tools such as guidelines. Here, doctors must proceed with the final decision and treatment assisted by AI-based risk assessment alone. Preemptive training is crucial for safe CDS use, especially with AI, as each tool will operate differently. Doctors need to understand what inputs the AI considers or excludes and be aware of how incorrect use could harm patients [42]. This study therefore highlights the need for AI developers and users to address safety considerations in AI-based CDS for resource-limited settings, to enhance global health equity rather than introduce new risks or burdens.

### Strengths and Limitations

This is the first randomized controlled study to evaluate the effects of AI on ASCVD risk assessment and patient management for primary prevention in a resource-constrained setting. Participants were instructed to perform the risk assessment as they would in a real outpatient setting, exercising their best clinical judgment. The awareness of completing clinical vignettes might have influenced doctors to perform at an enhanced level compared to real-world practice and overestimate improvement [17]. Conversely, doctors could assume that any inaccurate decision carried no accountable consequence.

We specifically designed the cases for resource-constrained clinical settings where there is access to information on traditional risk factors but limited access to advanced examinations such as computed tomography calcium scores, lipoprotein (a), and medical specialists. Our findings can therefore be generalized to similar settings. However, the spectrum within resource-constrained settings varies both horizontally across different dimensions and vertically within a single dimension [3]. For example, within the dimension of limited access to advanced examinations, even traditional risk factors like high-density lipoprotein cholesterol tests may be unavailable in some settings. Efforts were made to ensure that the cases were representative of the clinical context; however, this may not reflect the true rate of CVD risk in the population. Although the minimum sample size was determined a priori and was adequate, recruitment through the Indonesian Heart Association at 5 training centers may have led to a participant group more predisposed to research involvement than the broader medical community. Therefore, the findings may not be generalizable to the broader population of Indonesian GPs, who may be older, have different training backgrounds, and practice in more isolated community health centers (Table 1).

Additionally, we used a conceptual prototype of an AI-based CDS that accounted for REFs based on expert consensus, which may not reflect the true performance difference between an actual AI-based tool and the existing risk calculator. Actual AI-based tools have dynamic, rather than fixed, performance as they are continually trained on new data. In addition, the conceptual prototype did not account for practical challenges such as input data quality and model transportability across settings, which may limit the generalizability of the results to real-world settings. Nevertheless, this study has demonstrated the potential of AI-based CDS to improve risk assessment by accounting for patient-level precision beyond traditional risk factors and sets the foundation for further studies to test AI-based tools ahead of clinical implementation.

### Conclusions

Improvements in ASCVD risk assessment with a conceptual prototype demonstrate that use of AI can support better and faster decision-making in resource-constrained settings and are moderated by doctors’ positive perceptions.

## Supplementary material

10.2196/73131 Multimedia Appendix 1 Example patient case.

10.2196/73131 Multimedia Appendix 2 Results—a multilevel model for decision-making time.

10.2196/73131 Checklist 1 CONSORT checklist.

## Abbreviations

AI: artificial intelligence
ASCVD: atherosclerotic cardiovascular disease
CDS: clinical decision support
CONSORT: Consolidated Standards of Reporting Trials
CVD: cardiovascular disease
GP: general practitioner
ML: machine learning
REF: risk-enhancing factor
STROBE: Strengthening the Reporting of Observational Studies in Epidemiology
TAM: technology acceptance model
