# Supplementary material for: AI-Assisted Cardiovascular Risk Assessment by General Practitioners in Resource-Constrained Indonesian Settings Using a Conceptual Prototype: Randomized Controlled Study
Source: J Med Internet Res. 2025 Nov 25;27:e73131. doi: 10.2196/73131 (PMC12646556; doi:10.2196/73131)
Supplement: Multimedia Appendix 1 [file jmir-v27-e73131-s001.docx]

# Multimedia Appendix 1 – Example patient case

**Patient case**

|  | **English** | **Bahasa Indonesia** |
| --- | --- | --- |
| Identity | Mr. Ahmad, 65 years old, a retired civil servant | Bapak Ahmad, 65 tahun, pensiunan PNS |
| History taking | He come for routine diabetic drug dispensing, metformin. He was diagnosed with DM 3 years ago just before pandemic started. No hypertension. Never smoke since young. | Dia datang untuk mendapatkan obat diabetes. Dia didiagnosis dengan DM 3 tahun yang lalu sebelum pandemi COVID-19. Tidak ada riwayat hipertensi. Tidak pernah merokok sejak muda. |
| Physical examination | HR = 66x/min regular, BP = 130/80 mmHg. | Nadi = 66x/min regular, TD = 130/80 mmHg |
| Supporting examination | Total cholesterol = 190 mg/dL HDL = 60 mg/dL  Random blood glucose = 210 mg/dL | Total kolesterol = 190 mg/dL  HDL = 60 mg/dL  GDS = 210 mg/dL |

For each patient case, participants were asked to answer 6 compulsory questions (see below). The first two questions were asking the clinical decision about risk level and diagnosis. Questions number 3-5 were about prescriptions of aspirin, statin, and anti-hypertension.

Risk levels and recommendations of these prescriptions were explicitly provided in the Automated CDS and AI-based CDS but not in the control i.e. No decision support (see below). The last question was about referral decision to have an advanced examination for the patient provided this examination was only available at the nearest facility which is 1-3 hours away. The advanced examination included either treadmill stress test,[1] CT calcium score,[2] or CT coronary angiography.[3] There was no specific recommendation in CDS about this referral for advanced examination.

In all conditions, participants could access two national clinical guidelines on ASCVD prevention (see below).

**Questions and response options**

|  | **Question** | **Response options** |
| --- | --- | --- |
|  |  |  |
| Q1* | Apa estimasi stratifikasi risiko untuk kejadian PKVA dalam periode 10 tahun yang sesuai pada pasien ini?  *What is the estimate risk stratification of this patient having ASCVD events in 10-years time?* | 1. Low risk (<5%) 2. Borderline risk (≥5% - <7.5%) 3. Intermediate risk (≥7.5% - <20%) 4. High risk (≥20%) |
| Q2a* | Apakah diagnosis kerja pada pasien ini? Dapat memilih lebih dari satu pilihan  *What is your working diagnosis for this patient? You can choose more than one option* | 1. No applicable working diagnosis 2. Hypertension 3. DM type II (controlled or with hyperglycemia) 4. Hyperlipidemia |
| Q2b | Diagnosis kerja lainnya (jika ada)  *Additional working diagnosis (if any)* | *Free text* |
| Q3* | Dengan asumsi bahwa pasien ini tidak memiliki risiko perdarahan, apakah Anda akan berdiskusi dan meresepkan Aspirin 80-100 mg rutin pada pasien ini? *Assuming this patient does NOT have increased bleeding risk, will you discuss and prescribe routine aspirin 80-100 mg for this patient?* | 1. No 2. Yes, aspirin 80-100 mg |
| Q4* | Terapi rutin statin apakah yang Anda akan diskusikan dan resepkan pada pasien ini?  *What routine statin therapy will you discuss and prescribe for this patient?* | 1. No statin therapy 2. Low intensity statin   (e.g. Simvastatin 10-20 mg)   1. Moderate intensity statin   (e.g. Simvastatin 20-40 mg or Atorvastatin 10-20 mg)   1. High intensity statin   (e.g. Atorvastatin 40-80 mg or Rosuvastatin 20-40 mg) |
| Q5* | Terapi anti-hipertensi apakah yang akan anda diskusikan dan resepkan pada pasien ini?  *What anti-hypertensive therapy will you discuss and prescribe for this patient?* | 1. No anti-hypertensive therapy 2. Diuretic (e.g. Hydrochlorothiazide 25-50 mg) 3. CCB (e.g. Amlodipine 2.5-10 mg) 4. ACE-I (e.g. Captopril 12.5-150 mg bd or tds) or ARB (Candesartan 8-32 mg) 5. ACE-I or ARB + CCB 6. CCB + diuretic 7. ACE-I or ARB + diuretic |
| Q6a* | Jika fasilitas terdekat berada 1-3 jam perjalanan, apakah anda akan merujuk pasien untuk melakukan Exercise Stress Test atau CT Coronary Angiography? *If the nearest facility is 1-3 hours away, will you refer patient for Exercise Stress Test or CT Coronary Angiography?* | 1. No 2. Yes, exercise stress test or CTCA |
| Q6b | Jika iya, mohon jelaskan pertimbangan anda  *If yes, please state your consideration* | *Free text* |

**No decision support**

*Displaying Q1 to Q2b*


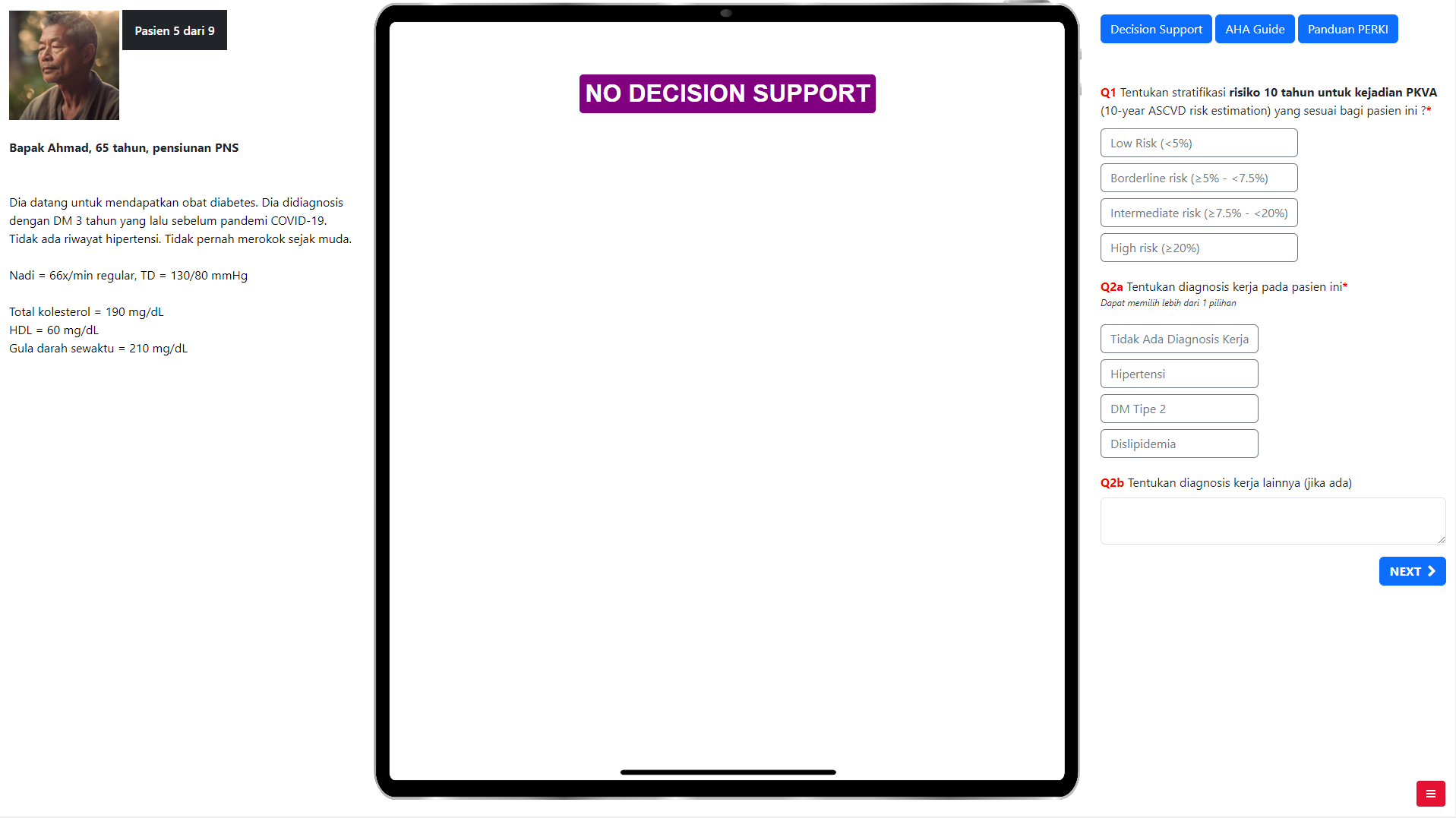


**No decision support – contd.**

*Displaying Q3 to Q6a*


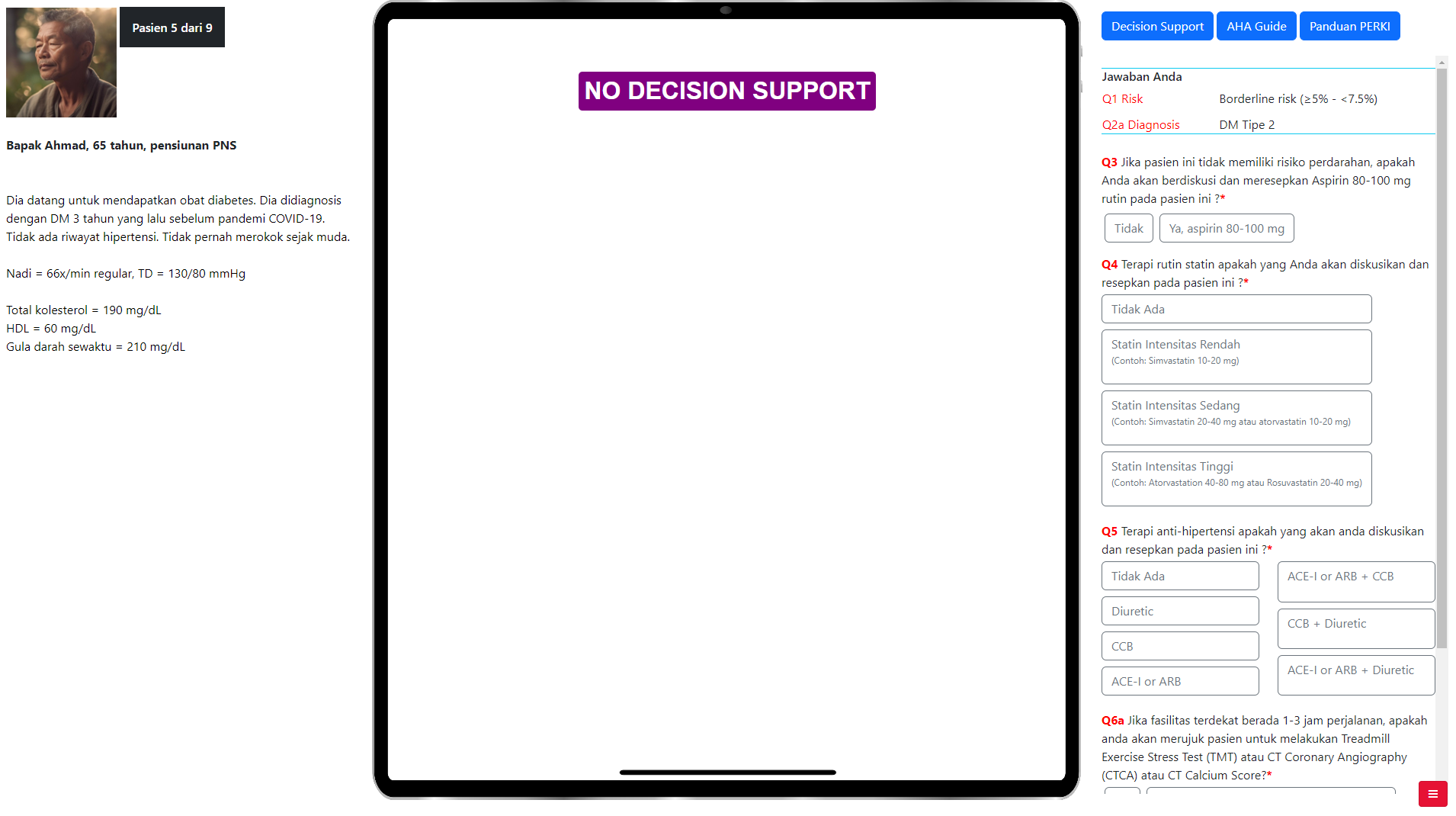


**Automated CDS**


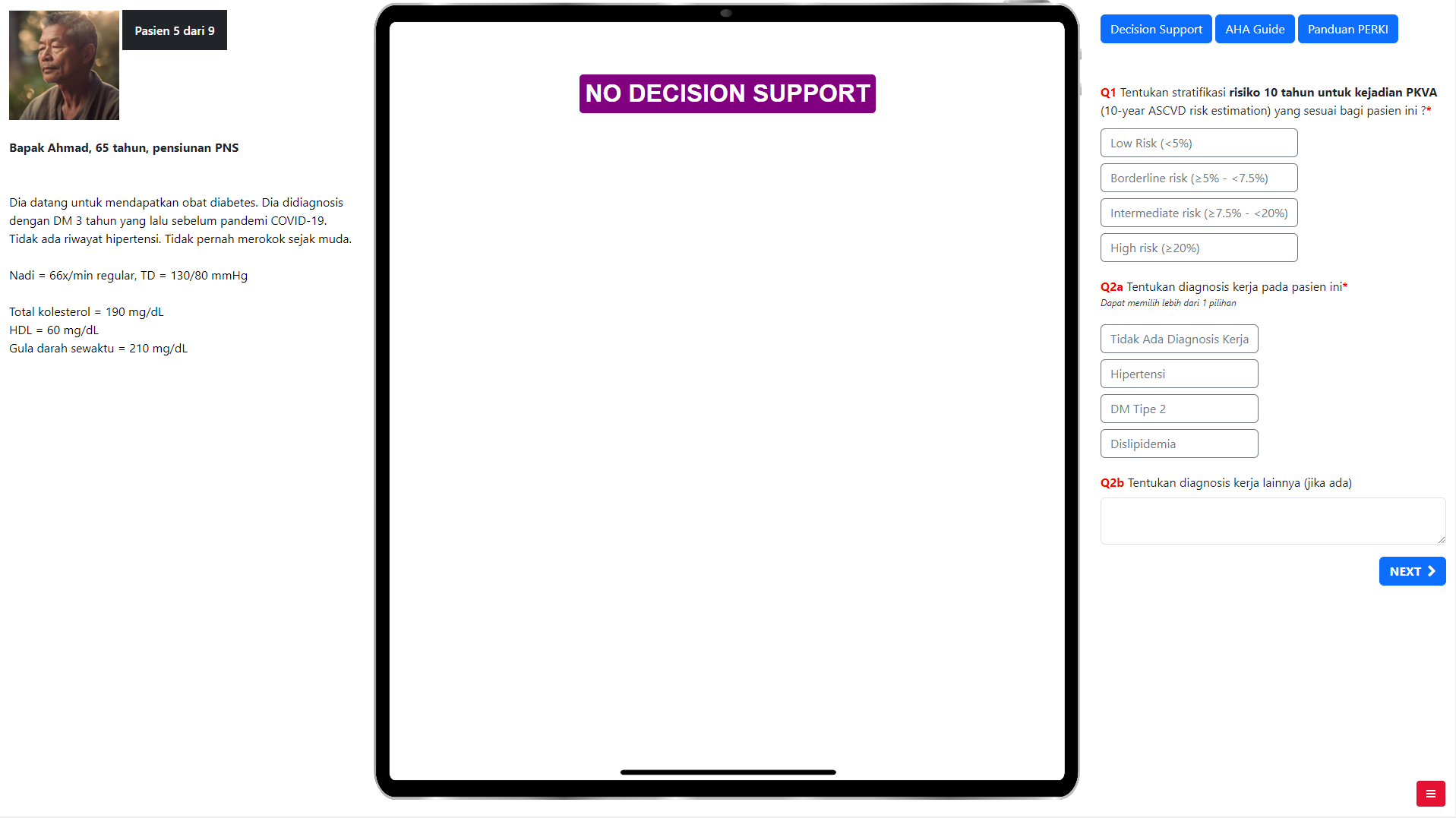

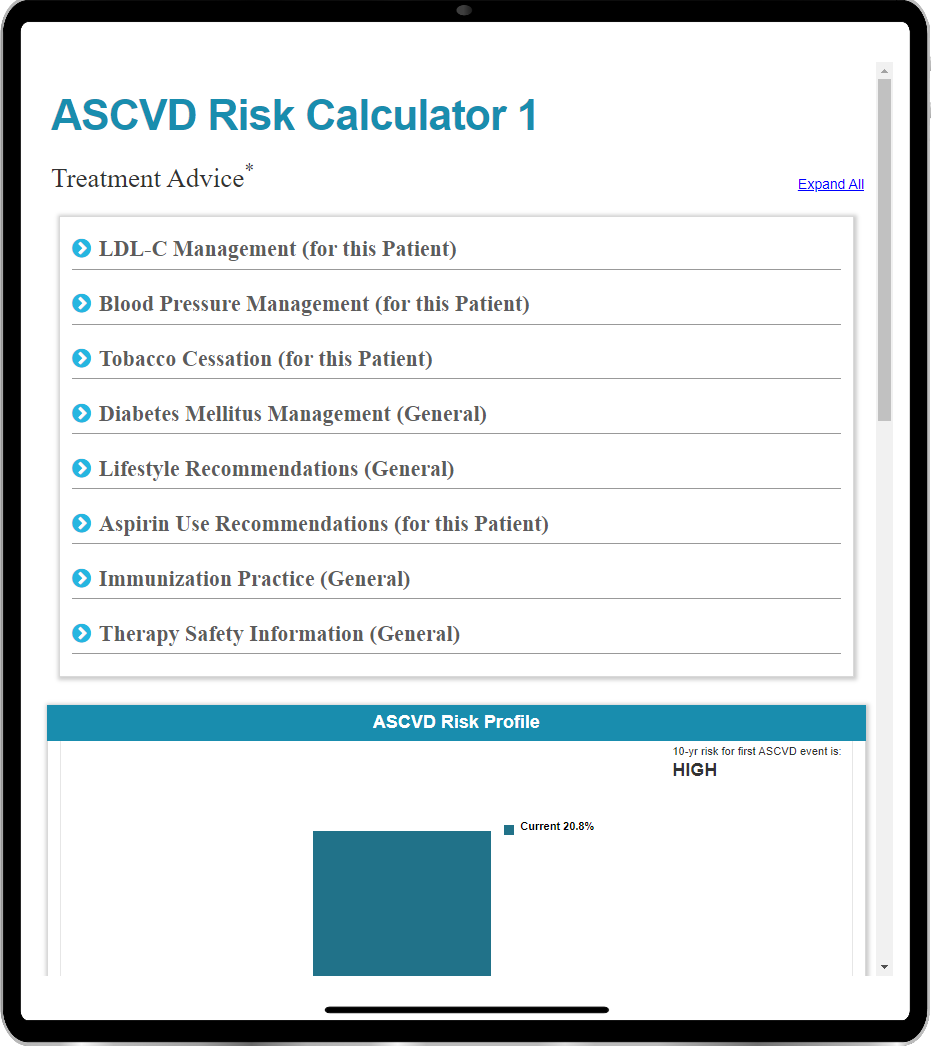


**AI-based CDS**


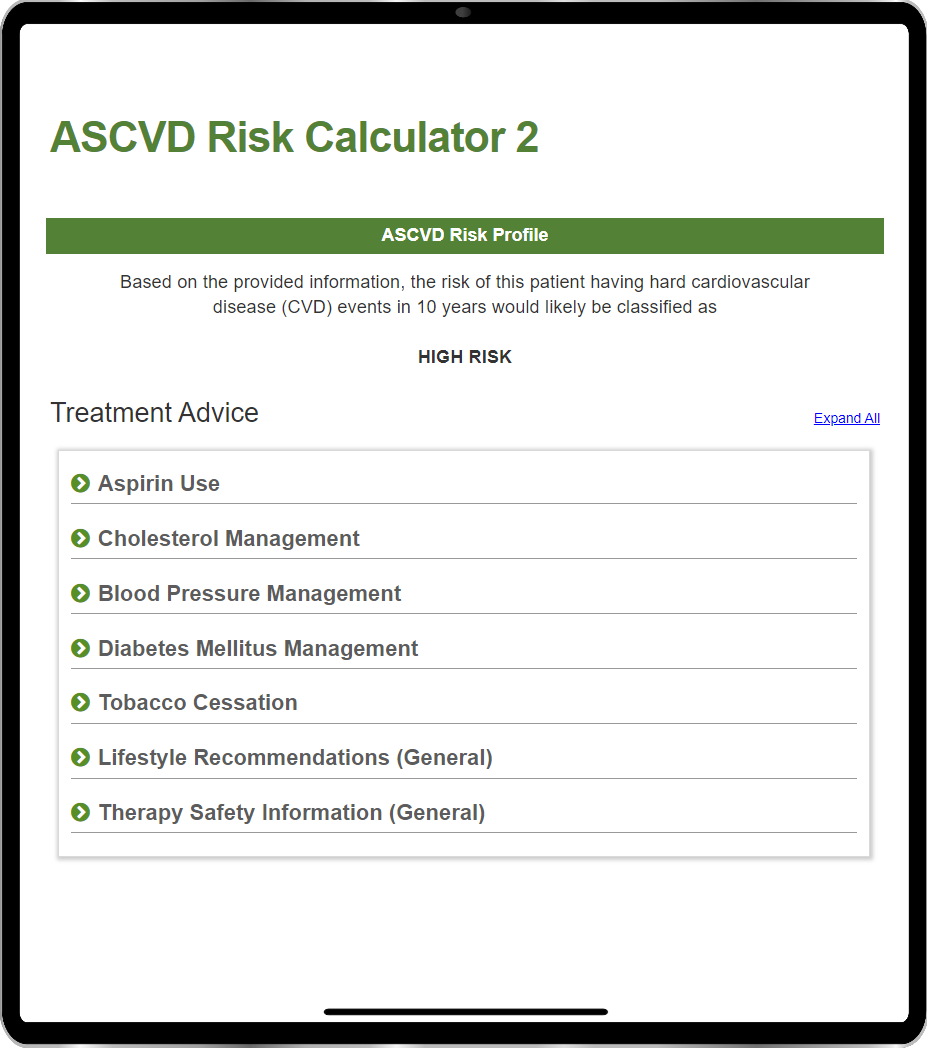

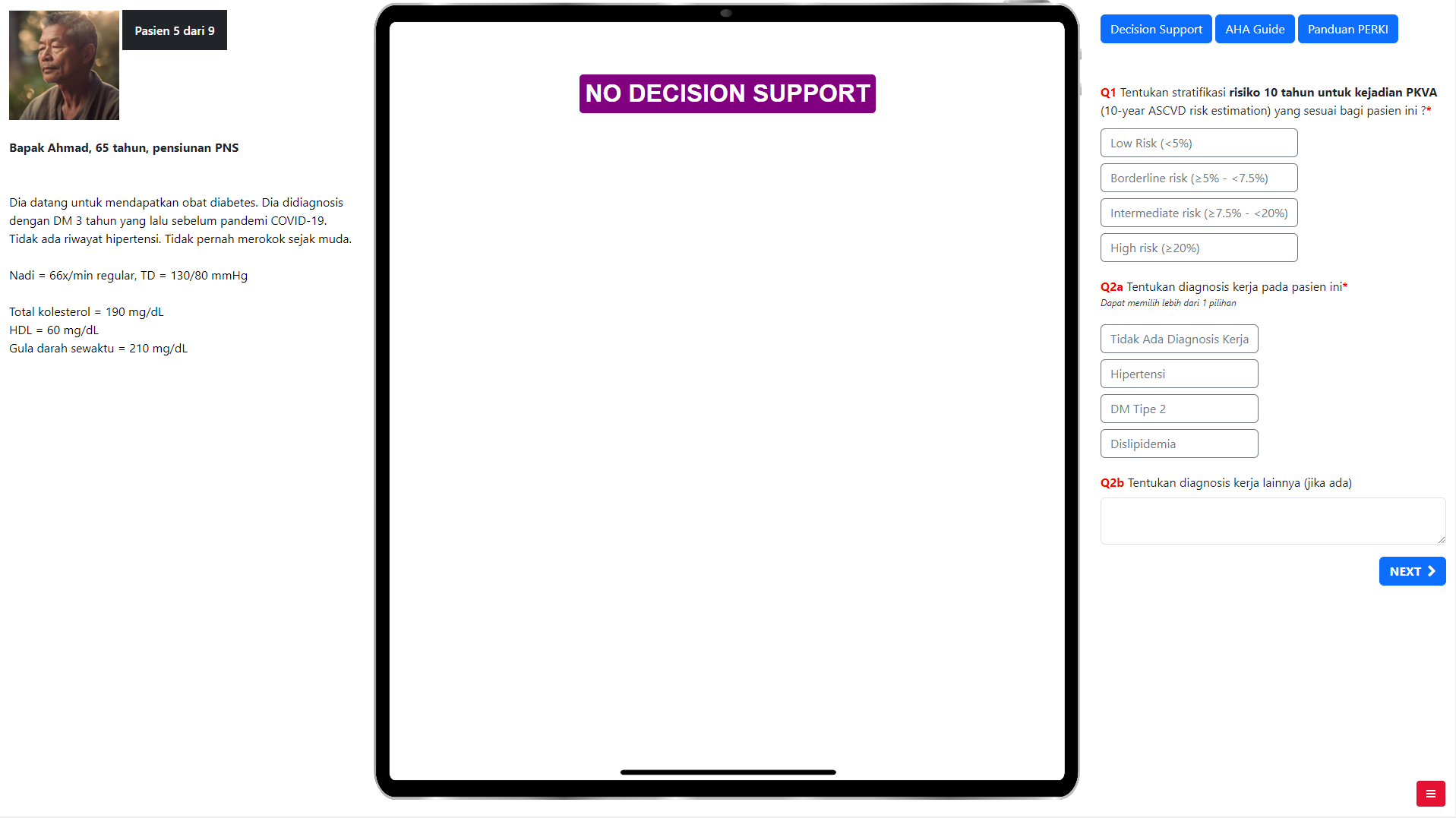


**Display of national clinical guidelines on ASCVD prevention – available for all CDS types**


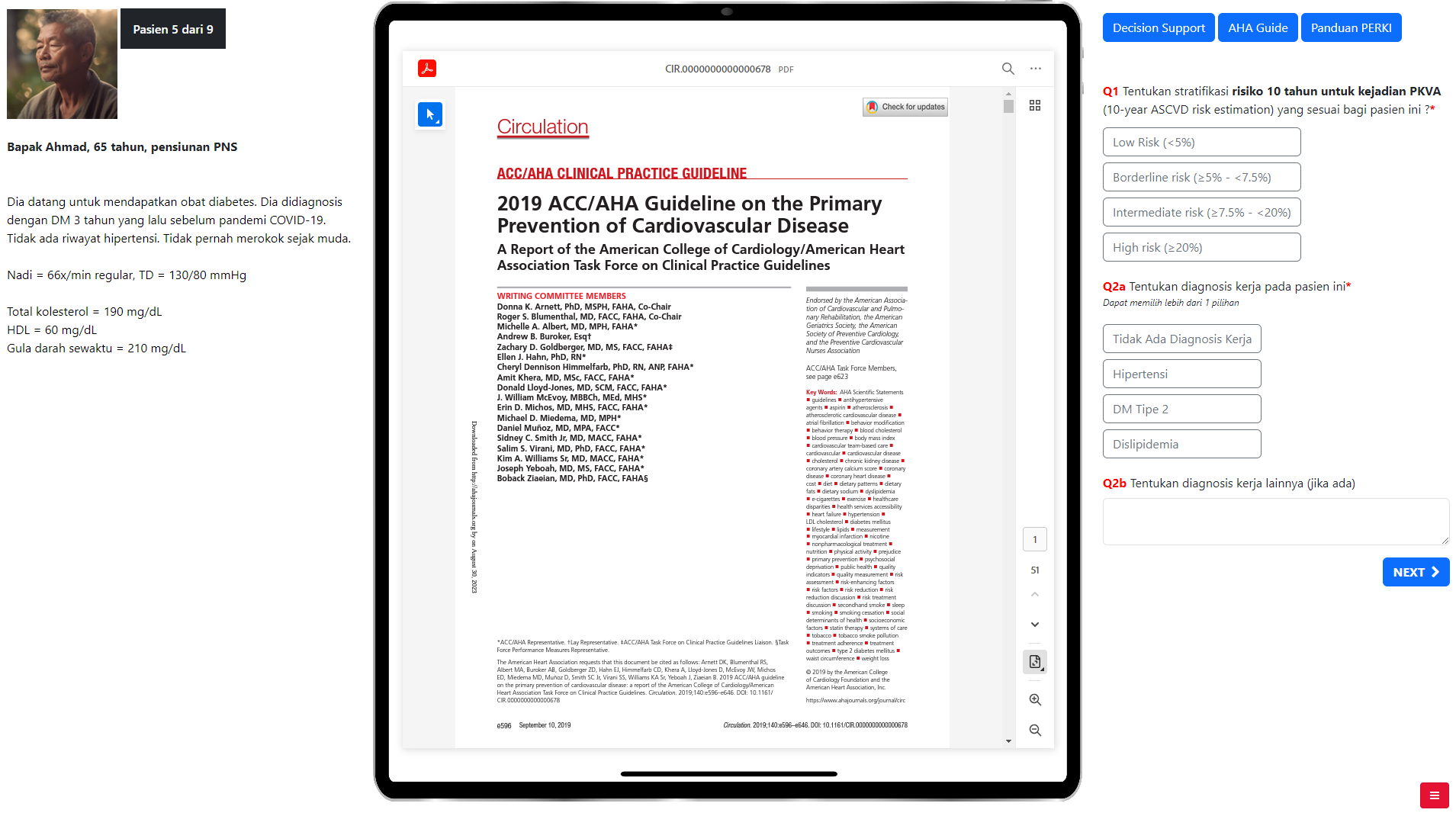


# References

1. Garner KK, Pomeroy W, Arnold JJ. Exercise stress testing: Indications and common questions. Am Fam Physician 2017;96:293-299.

2. Whelton SP, Nasir K, Blaha MJ, et al. Coronary artery calcium and primary prevention risk assessment: what is the evidence? An updated meta-analysis on patient and physician behavior. Circ Cardiovasc Qual Outcomes 2012;5(4):601-607.

3. Mortensen MB, Blaha MJ. Is There a Role of Coronary CTA in Primary Prevention? Current State and Future Directions. Curr Atheroscler Rep 2021;23(8):44.
