# Supplementary material for: AI-Assisted Cardiovascular Risk Assessment by General Practitioners in Resource-Constrained Indonesian Settings Using a Conceptual Prototype: Randomized Controlled Study
Source: J Med Internet Res. 2025 Nov 25;27:e73131. doi: 10.2196/73131 (PMC12646556; doi:10.2196/73131)
Supplement: Multimedia Appendix 2 [file jmir-v27-e73131-s002.docx]

# Multimedia Appendix 2 - Results

## A multilevel model for decision-making time.

The effect of quality of decision support (Automated or AI), scenario complexity, and participant cohort on decision-making time were analysed by a multilevel model.

Decision-making times were positively skewed (towards shorter times). Median decision-making time was 75 seconds (IQR = 93 seconds), with several substantial outliers, including two scenarios taking more than 4 hours and three scenarios taking more than 15 minutes but less than an hour. Outliers for decision-making time indicate the presence of meaningful interruptions. As such the measured time does not represent actual decision-making time. These outliers were, therefore, excluded from the analysis. The outlier labelling rule with a multiplier of 2.2 informed the identification of outliers, which suggests an upper limit of 334 seconds (5 mins 34 sec). A consultant cardiologist confirmed that 5 minutes was a reasonable upper limit to understand the case and decide. There were 45 outlier cases from 28 participants excluded from the analysis. Decision-making time for the remaining 873 cases (95.1%) included in the model, with a median of 69 seconds (IQR = 84 seconds).

We initially evaluated three models using decision-making time as the dependent variable however, the model residuals were not normally distributed. The decision time was then computed using a logarithmic scale. We evaluated a full model, and from this, two out of three fixed effects were found to contribute significantly to the fit of a multilevel model. This was the final model with the case complexity (*p* < 0.001), and the quality of decision support (*p* = 0.003) was found to be statistically significant. However, the interaction between case complexity and quality of decision (*p* = 0.488) was not found to be significant but still included in the model due to theoretical importance. The estimates of fixed effects and the model coefficients are presented in Table S3.2. The effects are compared based on the estimated marginal means computed by the model. Significance probabilities have been adjusted for multiple comparisons using the Bonferroni correction. The final model was significantly better than the null model (χ^2^(8) = 72.932; *p* < 0.001). The intraclass correlation coefficient was 0.484, indicating that 48% of the variance in decision-making time was attributable to variation between participants, supporting the conduct of a multilevel analysis. The model residuals were normally distributed. We then converted backlog values of decision-making time to visualise the estimated marginal mean (EMM).

Table S4.2 Estimates of fixed effects in the multilevel model of decision time (Log)

| **Source** | **df** | **F** | ***p*-Value** |
| --- | --- | --- | --- |
| Intercept | 1, 102.185 | 4976.971 | <.001* |
| Case complexity | 2, 772.507 | 30.725 | <.001* |
| Quality of decision support | 2, 772.800 | 5.710 | .003* |
| Case complexity x quality of decision support | 4, 772.387 | .859 | .488 |

High complexity cases took significantly longer to assess (F(2, 772.5)=30.725, *p*<0.001). Doctors spent significantly more time on high complexity cases with REF (EMM=74.1 sec; 95% CI 65.0 – 84.4; *p*<0.001) and high complexity cases (EMM=73.6 sec; 95% CI 64.6–83.8; *p*<0.001) compared to low complexity cases (EMM=53.2 sec; 95% CI 46.7–60.52). There was no significant difference in the decision-making time between the high complexity cases with and without the REF.

Table S4.3: Selection of predictors for multilevel model (Logarithm of decision-making time)

| **Model evaluated** | **Fixed effect removed** | **-2 Log Likelihood** | **Number of parameters** | **Likelihood ratio test** | **Include in final model** |
| --- | --- | --- | --- | --- | --- |
| **[Full & Final model]** Fixed intercept, random intercept (participant), case complexity, quality of decision support, quality*complexity |  | 305.721 | 11 |  | Include |
| **[Intercepts only model]** Fixed intercept, random intercept (participant) | Case complexity, quality of decision support, quality*complexity | 378.653 | 3 | χ2(8)=72.932 *p*=.000 | Include |
| Fixed intercept | Random intercept (participant) | 698.6 | 2 | χ2(1)=319.947 *p*=.000 | Include |
| **[Null model]** | Fixed intercept | 3551.586 | 1 | χ2(1)=2852.986 *p*=.000 | Include |

Table S4.4: Multilevel model coefficients for the logarithm of decision-making time

| **Parameter** | **Coefficient** | **Std Error** | **df** | **t** | **Sig.** | **95% Conf. Interval** | |
| --- | --- | --- | --- | --- | --- | --- | --- |
|  |  |  |  |  |  | **Lower Bound** | **Upper Bound** |
| Intercept | 1.847274 | .035584 | 332.788 | 51.913 | .000 | 1.777276 | 1.917271 |
| Low complexity | -.142881 | .036424 | 772.533 | -3.923 | .000 | -.214383 | -.071379 |
| High complexity | .011426 | .036325 | 772.441 | .315 | .753 | -.059881 | .082734 |
| High complexity with REF | 0 | 0 | . | . | . | . | . |
| Control (No CDS) | .046181 | .036745 | 773.136 | 1.257 | .209 | -.025951 | .118314 |
| Automated CDS | .021051 | .036590 | 772.344 | .575 | .565 | -.050776 | .092877 |
| AI-based CDS | 0 | 0 | . | . | . | . | . |
| Low complexity * control (No CDS) | .043264 | .051660 | 772.687 | .837 | .403 | -.058146 | .144674 |
| Low complexity * automated CDS | -.046660 | .051424 | 772.401 | -.907 | .365 | -.147608 | .054288 |
| Low complexity * AI-based CDS | 0 | 0 | . | . | . | . | . |
| High complexity * control (No CDS) | -.006209 | .051961 | 772.806 | -.119 | .905 | -.108212 | .095793 |
| High complexity * automated CDS | -.036681 | .051483 | 772.318 | -.712 | .476 | -.137745 | .064383 |
| High complexity * AI-based CDS | 0 | 0 | . | . | . | . | . |
| High complexity with REF * control (No CDS) | 0 | 0 | . | . | . | . | . |
| High complexity with REF * automated CDS | 0 | 0 | . | . | . | . | . |
| High complexity with REF * AI-based CDS | 0 | 0 | . | . | . | . | . |

**References for Multimedia Appendix**

1. Garner KK, Pomeroy W, Arnold JJ. Exercise stress testing: Indications and common questions. Am Fam Physician 2017;96:293-299.

2. Whelton SP, Nasir K, Blaha MJ, et al. Coronary artery calcium and primary prevention risk assessment: what is the evidence? An updated meta-analysis on patient and physician behavior. Circ Cardiovasc Qual Outcomes 2012;5(4):601-607.

3. Mortensen MB, Blaha MJ. Is There a Role of Coronary CTA in Primary Prevention? Current State and Future Directions. Curr Atheroscler Rep 2021;23(8):44.

4. Cheng A, Kessler D, Mackinnon R, et al. Reporting guidelines for health care simulation research: extensions to the CONSORT and STROBE statements. Adv Simul (Lond) 2016;1:25.
