# Supplementary material for: AI-Assisted Cardiovascular Risk Assessment by General Practitioners in Resource-Constrained Indonesian Settings Using a Conceptual Prototype: Randomized Controlled Study
Source: J Med Internet Res. 2025 Nov 25;27:e73131. doi: 10.2196/73131 (PMC12646556; doi:10.2196/73131)
Supplement: Checklist 1 [file jmir-v27-e73131-s003.docx]

# CONSORT checklist

**Simulation-based research extensions for the CONSORT Statement (**Cheng, 2016).

| **Item** | **No.** | **CONSORT description (Randomised Controlled Trials)** | **Extensions for SBR** | **This article** |
| --- | --- | --- | --- | --- |
| Title and abstract | 1 | 1. Identification as a randomised trial in the title 2. Structured summary of trial design, methods, results, and conclusion | In abstract of key terms, the MESH or searchable keyword term must have the word “simulation” or “simulated” | Subtitle: **a randomised controlled simulation** study with Indonesian doctors  See ‘Abstract’ section |
| Introduction | 2 | 1. Scientific background and explanation of rationale 2. Specific objectives or hypothesis | Clarify whether simulation is subject of research or investigational method for research | See ‘Introduction’ section. |
| **Methods** |  |  |  |  |
| Trial design | 3 | 1. Description of trial design (such as parallel factorial) including allocation ratio 2. Important changes to methods after trial commencement (such as eligibility criteria), with reasons |  | See ‘Trial design’ |
| Participants | 4 | 1. Eligibility criteria for participants 2. Settings and locations where the data were collected |  | See ‘Setting and participants’ |
| Interventions | 5 | The interventions for each group with sufficient details to allow for replication, including how and when they were actually administered | Describe the theoretical and/or conceptual rationale for the design of each intervention. Clearly describes all simulation-specific exposures, potential confounders, and effect modifiers. | See ‘Interventions’ |
| Outcomes | 6 | 1. Completely defined prespecified primary and secondary outcome measures, including how and when they were assessed. 2. Any changes to trial outcomes after the trial commenced, with reasons | In describing the details of methods of assessment, include (when applicable) the setting, instrument, simulator type, timing in relation to the intervention, along with any methods used to enhance the quality of measurements. Provide evidence to support the validity and reliability of assessment tools in this context (if available) | See ‘Outcome measures’  No changes to trial outcomes. |
| Sample size/ study size | 7 | 1. How ample size was determined 2. When applicable, explanation of any interim analyses and stopping guidelines |  | See ‘Statistical analysis.’ |
| Randomisation: sequence generation | 8 | 1. Method used to generate the random allocation sequence 2. Type of randomization and details of any restriction (such as blocking and block size) |  | See ‘trial design’ and ‘procedure’ |
| Randomization: allocation concealment mechanism | 9 | Mechanism used to implement the random allocation sequence (such as sequentially numbered containers), describing any steps taken to conceal the sequence until interventions were assigned. |  | See ‘trial design’ and ‘procedure’ |
| Randomization: implementation | 10 | Who generated the random allocation sequence, who enrolled participants, and who assigned participants to interventions |  | See ‘trial design’ and ‘procedure’ |
| Blinding (masking) | 11 | 1. If done, who was blinded after assignments to interventions (e.g. participants, care providers, those assessing outcomes) and how 2. If relevant, description of the similarity of interventions | Describe strategies to decrease risk of bias, when blinding is not possible | See ‘interventions’ |
| Statistical methods | 12 | 1. Statistical methods used to compare groups for primary and secondary outcomes 2. Methods for additional analyses, such as subgroup analyses and adjusted analyses | Clearly indicate the unit of analysis (e.g. individual, team, system), identify repeated measures on subjects, and describe how these issues were addressed. | See ‘Statistical analysis’ |
| **Results** |  |  |  |  |
| Participant flow (a diagram is strongly recommended) | 13 | 1. For each group, the numbers of participants who were randomly assigned, received intended treatment, and were analysed for the primary outcome. 2. For each group, losses and exclusions after randomization, together with reasons. |  | See ‘Multimedia Appendix 3’ |
| Recruitment | 14 | 1. Dates defining the periods of recruitment and follow-up 2. Why the trial ended or was stopped |  | See ‘Setting and participants’ |
| Baseline data | 15 | A table showing baseline demographic and clinical characteristics of each group | In describing characteristics of study participants, include their previous experience with simulation and other relevant features as related to the intervention(s) | See ‘Table 1’ and ‘Multimedia Appendix 3’ |
| Number analysed | 16 | For each group, number of participants (denominator) included in each analysis and whether analysis was by original assigned groups |  | See ‘Figure 1’ |
| Outcomes and estimation | 17 | 1. For each primary and secondary outcome, results for each group, and the estimated effect size and its precision (such as 95% confidence interval) 2. For binary outcomes, presentation of both absolute and relative effect sizes is recommended | For assessments involving > 1 rater, interrater reliability should be reported | See ‘Results’, ‘Table 2’, and ‘Multimedia Appendix 4’ |
| Ancillary analyses | 18 | Results of any other analyses performed, including subgroup analyses and adjusted analyses, distinguishing prespecified from exploratory |  | Not available |
| Adverse events | 19 | All important harms or unintended effects in each group (for specific guidance, see CONSORT for harms) |  | Not available |
| **Discussion** |  |  |  |  |
| Limitations | 20 | Trial limitations, addressing sources of potential bias, imprecision, and if relevant, multiplicity of analyses | Specifically discuss the limitations of SBR |  |
| Generalizability | 21 | Generalizability (external validity, applicability) of the trial findings | Describe generalizability of simulation-based outcomes to patient-based outcomes (if applicable) | See ‘Strength and limitation’ |
| Interpretation | 22 | Interpretation consistent with results, balancing benefits and harms, and considering other relevant evidence |  | See ‘Main findings and implications’ |
| **Other information** |  |  |  |  |
| Registration | 23 | Registration number and name of trial registry |  | Not available |
| Protocol | 24 | Where the full trial protocol can be accessed, if available |  | See ‘Method’ and ‘Multimedia Appendix ’ |
| Funding | 25 | Sources of funding and other support (such as supply of drugs), role of funders | List simulator brand and if conflict of interest for intellectual property exists | See ‘Funding’ |

**Key elements to report for simulation-based research**

| **Elements** | **Subelements** | **Descriptor** | **This article** |
| --- | --- | --- | --- |
| Participant orientation | Orientation to the simulator | Describe how participants were oriented to the simulator (e.g., method, content, duration) | See ‘Procedure’ |
|  | Orientation to the environment | Descirbe how participants were oriented to the environment (e.g. method, content, duration) | See ‘Procedure’ |
| Simulator type | Simulator make and model | Describe the simulator make and model | See ‘Trial design’ |
|  | Simulator functionality | Describe functionality and/or technical specifications that are relevant to the research question. Describe modifications, if any. Describe limitations of the simulator. | See ‘Trial design’ |
| Simulation environment | Location | Describe where the simulation was conducted (e.g. in situ clinical environment, simulation center, etc.) | See ‘Procedure’ |
|  | Equipment | Describe any external stimuli (e.g. background noise) | See ‘Procedure’ |
|  | External stimuli | Describe any external stimuli (e.g. background noise) | See ‘Procedure’ |
| Simulation event/ scenario | Event description | Describe if the event was programmed and/or scripted (e.g. orientation to event, scenario progression, triggers). If a scenario was used, the scenario script should be provided as an appendix. | See ‘Procedure’ |
|  | Learning objectives | List the learning objectives and describe how they were incorporated into the event. | See ‘Procedure’ |
|  | Group vs individual practice | Describe if the simulation was conducted in groups or as individuals | See ‘Procedure’ |
|  | Use of adjuncts | Describe if adjuncts (e.g. moulage, media, props) were used | Not available |
|  | Facilitator/operator characteristics | Describe experience (e.g. clinical, educational), training (e.g. fellowships, courses), profession. | Not available |
|  | Pilot testing | Describe if pilot testing was conducted (e.g. number, duration, frequency). | See ‘Procedure’ |
|  | Actors/confederates/ standardised/ simulated patients | Describe experience (e.g. clinical, educational), profession, sex. Describe various roles, including training, scripting, orientation, and compliance with roles. | Not available |
| Instructional design (for educational interventions) or exposure (for simulation as investigative methodology) | Duration | Describe the duration of the educational intervention. If the intervention involves more than one segment, describe the duration of each segment. | See ‘Procedure’ |
|  | Timing | Describe the timing of the educational intervention relative to the time when assessment/data collection occurs (e.g. just-in-time training) | See ‘Procedure’ |
|  | Frequency/repetitions | Describe how many repetitions were permitted and/or the frequency of training (e.g. deliberate practice) | See ‘Procedure’ |
|  | Clinical variation | Describe the variation in clinical context (e.g. multiple different patient scenarios) | See ‘Trial design’ |
|  | Standard/assessment | Describe predefined standards for participant performance (e.g. mastery learning) and how these standards were established. | See ‘Outcome measures’ |
|  | Adaptability of intervention | Describe how the training was responsive to individual learner needs (e.g. individualized learning) | Not available |
|  | Range of difficulty | Describe the variation in difficulty or complexity of the task | See ‘Trial design’ |
|  | Nonsimulation interventions and adjuncts | Describe all other nonsimulation interventions (e.g. lecture, small group discussion) or educational adjuncts (e.g. educational video), how they were used, and when they were used relative to the simulation intervention. | Not available |
|  | Integration | Describe how the intervention was integrated into curriculum | Not available |
| Feedback and/or debriefing | Source | Describe the source of feedback (e.g. computer, simulator, facilitator) | See ‘Procedure’ |
|  | Duration | Describe the among of time spent | Not available |
|  | Facilitator presence | Describe if a facilitator was present (yes/no), and if so, how many facilitators | Not available |
|  | Facilitator characteristics | Describe experience (e.g. clinical, educational), training (e.g. fellowship, courses), profession, sex. | Not available |
|  | Content | Describe content (e.g. teamwork, clinical, technical skills, and/or inclusion of quantitative data, etc.) | Not available |
|  | Structure/method | Describe the method of debriefing/feedback and debriefing framework used (i.e., phases) | Not available |
|  | Timing | Describe when the feedback and/or debriefing was conducted relative to the simujaltion event (e.g. terminal vs concurrent) | See ‘Procedure’ |
|  | Video | Describe if video was used (yes/no) and how it was used | Not available |
|  | Scripting | Describe if a script was used (yes/no) and provide script details as an appendix. | Not available |
